# P16 Strengthening surveillance systems for antimicrobial resistance in urinary tract infections in Kenya

**DOI:** 10.1093/jacamr/dlac053.016

**Published:** 2022-05-31

**Authors:** G Bartonjo, E Ngata, G Revathi, A Aiken

**Affiliations:** 1 National Public Health Laboratory, Nairobi, Kenya; 2 Aga Khan University Hospital, Nairobi, Kenya; 3 London School of Hygiene &Tropical Medicine, London, UK

## Abstract

**Background:**

Urinary tract infections (UTIs) are amongst the most common conditions leading to outpatient visits and are also one of the most common causes of inappropriate antibiotic usage. Addressing antimicrobial resistance (AMR) requires strengthening surveillance system in Kenya where UTI is a problem with health systems having limited capacity for accurate laboratory identification of UTI pathogens. Empirical treatment to manage UTIs is the most common problem due to limited microbiology capacity. These include shortages of suitable training, equipment, quality control procedures and professional expertise to make an accurate clinical interpretation of urine cultures. This leads to reporting normal flora as pathogens.

**Objectives:**

To deliver training on the identification and antibiotic susceptibility testing of bacterial isolates from urine infections at participating hospitals in the Kenyan AMR surveillance network and measure competency levels in staff participating in the training with the aim of strengthening the Kenyan surveillance system.

**Methods:**

A 1 day baseline visit to the five sites was made between October and November 2021 using a standardized checklist to assess laboratory capacity. This was followed by urine processing training conducted between November 2021 and January 2022 in Malindi, Jaramogi, Kitale, Nakuru and Nyeri County Hospitals. Training materials were developed with the input of Aga Khan University Hospital. A 5 day in-person training course was delivered at each of the five hospitals, which targeted an average of 2–3 laboratory staff per hospital. The competencies of the laboratory staff were assessed by written pre-assessment and post-assessment tests. A standardized practical assessment was achieved by use of five different control organisms. The scores were done by doing an average performance score at each hospital laboratory.

**Results:**

Out of 13 trainees, 8 were male and 5 were female. The median number of years since qualification for these staff was 6 (IQR 2–9). The median number of total months of microbiology training time was 2.3 (IQR 1–7). The median pre-training and post-training scores were 46% and 90%, respectively; non-parametric Wilcoxon rank test showed statistical difference (*P* < 0.0001) between pre-training and post-training. All participants improved from pre-to post- training assessment from the 5 day training with marked improvement amongst from staff with lowest previous level of laboratory training.

**Conclusions:**

Training microbiology staff in the accurate processing of urine sample will be an important activity for a Kenyan AMR surveillance system. These training materials, if delivered by an experienced trainer, can achieve a clear improvement in knowledge levels and practical competence for real-life microbiology identifications.

